# Colonization of *Panstrongylus megistus* (Hemiptera:Reduvidae:Triatominae) in an urban area and its association with *Didelphis marsupialis* in the metropolitan region of São Paulo

**DOI:** 10.1590/0037-8682-0471-2020

**Published:** 2021-02-26

**Authors:** Rubens Antonio da Silva, Raquel Zaicaner, Mariangela Palma Rosa, Graciela Cristina Granizo Aun, Júlio César Muniz, Alon Carlos Magalhães, Creusa Pereira, Amanda Rosa Pena, Maysa Flexa dos Santos, Vera Aparecida Oliveira Estevão, Agnaldo Nepomuceno Duarte

**Affiliations:** 1 Secretaria de Estado da Saúde de São Paulo, Superintendência de Controle de Endemias, Laboratório Especializado de Mogi Guaçu: Triatomíneos, São Paulo, SP, Brasil.; 2 Prefeitura de Taboão da Serra, Secretaria Municipal de Saúde, Taboão da Serra, SP, Brasil.; 3 Prefeitura de Taboão da Serra, Secretaria Municipal de Saúde, Diretoria de Vigilância em Saúde, Taboão da Serra, SP, Brasil.; 4 Prefeitura de Taboão da Serra, Secretaria Municipal de Saúde, Diretoria do Centro de Controle de Zoonoses, Taboão da Serra, SP, Brasil.; 5 Prefeitura de Taboão da Serra, Secretaria Municipal de Saúde, Centro de Controle de Zoonoses, Taboão da Serra, SP, Brasil.; 6 Secretaria de Estado da Saúde de São Paulo, Superintendência de Controle de Endemias, Centro Regional de São Paulo, São Paulo, SP, Brasil.

**Keywords:** Chagas disease, Panstrongylus sp., Urban area

## Abstract

**INTRODUCTION:**

This communication reports the colonization of *Panstrongylus megistus* in an urban area of the municipality of Taboão da Serra in the metropolitan region of São Paulo.

**METHODS:**

After receiving a notification from the population, entomological research comprising active search, collection, identification, and examination of triatomines was conducted. Wild animals were captured and examined.

**RESULTS:**

A colony of triatomines was found to be associated with dogs in the backyard of the property.

**CONCLUSIONS:**

The colonization of *P. megistus* shows the potential for their occupation of artificial ecotopes, which may pose a risk to the human population.

Chagas disease is a non-contagious infectious disease caused by a protozoan parasite called *Trypanosoma cruzi* and transmitted (in vector form) in the feces of a triatomine bug that is popularly known in Brazil as *barbeiro*. In São Paulo, vector transmission control was initiated in 1950, with actions targeted at *Triatoma infestans*, the main vector species. With satisfactory results in the home environment, this species was controlled and the triatomines more frequently collected in the peridomicile started to gain importance^1^ ^,^ ^2^. In this context, *Panstrongylus megistus* is considered to be the most important because of its association with humans, presence in the intradomiciliary environment, and high indices of natural infection^3^. The high infection indices found for the species is because they mainly use the blood of rodents and opossums as food sources^4^.

In Brazil, *P. megistus* is well distributed and its behavior is similar to that found in São Paulo: wide geographical distribution, high infection rates by *T. cruzi*, and a remarkable capacity to colonize artificial environments. They are less aggressive, and they preferentially feed on vertebrate blood. They do not fly large distances and are commonly transported in bird feathers. They usually take shelter in places that are in close proximity to the food source^4^. 

The metropolitan region of São Paulo (MRSP) comprises 39 municipalities, Taboão da Serra being one of them. Triatomines occurred sporadically in the municipality, with high rates of infection by *T. cruzi*, but with no evidence of colonization. In 2018, the first outbreak of the species *P. megistus* was found in the municipality of Carapicuíba, part of the MRSP, in a house located in an urban condominium very close to a forest and associated with a marsupial nest^5^. The objective of this study is to report, for the first time, the occurrence of a focus of *P. megistus* in the municipality of Taboão da Serra, in an urban area. 

The municipality of Taboão da Serra is located in the southwestern zone of the MRSP, with a population of 285,570 inhabitants and an area of 20.39 km^2^ (Figure 1)^6^. The municipality’s demographic density is 14,006.77 inhabitants/km^2^, and it is located at a 14-km distance from the State’s capital. It has approximately 79 hectares of Atlantic Forest fragments, average annual rainfall and temperature of 122.08 mm and 18.2°C, respectively, and an altitude of 736 m above sea level. 

**FIGURE 1:**
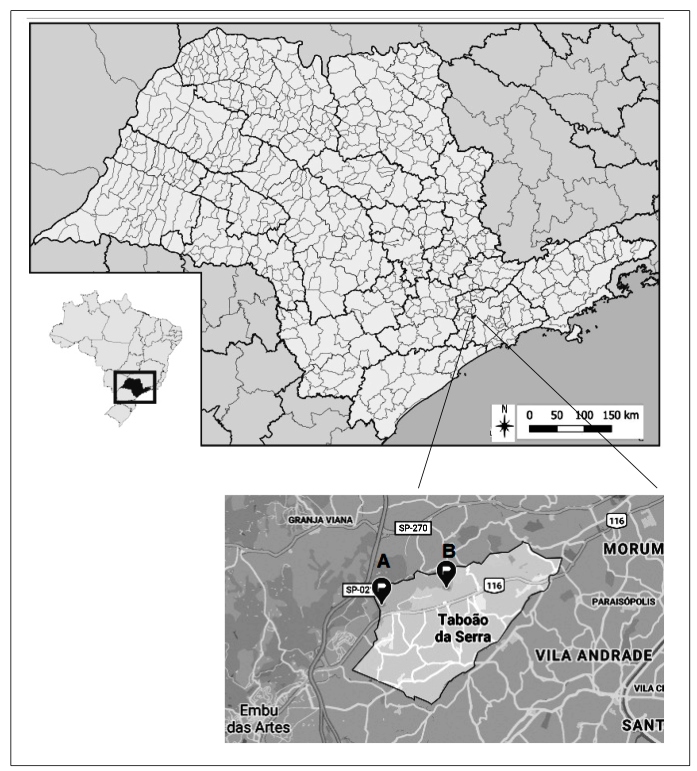
Municipality of Taboão da Serra. **(A)** point of constant invasion of triatomines and **(B)** point of colonization of *Panstrongylus megistus*.

Entomological surveillance in the state of São Paulo is conducted by encouraging the population to send specimens of insects suspected of being triatomines for evaluation^3^. The samples sent are identified as triatomines by examining the intestinal contents to check for trypanosomatid infections. If positive, the slides are prepared and stained to identify the parasite. In addition, a sample of the intestinal contents is collected on filter paper for further characterization of the eating habits. An encounter with a triatomine triggers thorough entomological research carried out by trained field teams in the notifying house and in those surrounding it. Upon receiving a triatomine notification, the response takes no more than 30 days from the date in which the insect was captured. The entomological research aims to find new specimens and/or vestiges and explores the house and the peridomiciliary area. It focuses on locations which are places of rest and shelter for animals that constitute these insects’ food source.

This activity involves the dweller or the person responsible for the dwelling, who also receive orientations regarding the places where the triatomine bugs are more likely to be found, so that they can take appropriate action to hinder the formation of vector colonies while also notifying its presence when found. When new triatomine specimens are found in the house, chemical control is performed with the use of pyrethroid insecticides, integrally in the peridomiciliary and selectively in the intradomiciliary areas.

To identify the circulation of *T. cruzi* infection in *Didelphis* spp., tomahawk traps were set in forest fragments near the residences. The captured animals were anesthetized, and their blood samples were collected for direct examination and blood culturing. In addition, they were subjected to a xenodiagnosis with the use of 10 fourth-instar nymphs of *T. infestans,* reared in the laboratory and biologically synchronized, which remained in contact with the animals’ skin for twenty minutes to feed on them. This project was submitted to and approved by two institutions: the Chico Mendes Institute for Biodiversity Conservation (ICMBio), under number 64775-1, and the Animal Ethics Committee of the Superintendence for Endemics Control, under number 0001/2019.

In 2016, in Taboão da Serra, *P. megistus* invaded well-built houses belonging to a condominium located in an Atlantic Forest reserve (Figure 1 - Point A) (23°36′57.1′′ S, 46°48′35.6′′ W). From notifications routinely made by dwellers, the presence of this vector was found, but there was no colonization by the species. Triatomine specimens (95) were collected in the municipality and 42.5% of them tested positive for *T. cruzi* (Table 1). 

**TABLE 1:** Number of notifications of triatomines of the species *Panstrongylus megistus* and specimens captured, examined, and tested positive, according to year; Taboão da Serra, 2016 to 2020*.

Year	No. of	Specimens			
	notifications	Captured	Examined	Positive	% Positive
2016	7	7	7	2	28.6
2017	25	25	25	6	24.0
2018	24	24	23	17	73.9
2019	21	34	34	12	35.3
2020*	6	5	5	3	60.0
Total	83	95	94	40	42.5

*Up to 05/31/2020.

In December 2019, a dweller notified the finding of two triatomines (one male adult that tested positive for *T. cruzi* and one fifth-instar nymph) in the backyard of his property located in the urban area of the municipality (Figure 1 - Point B). The municipal team that visited the dwelling in response to the notification located a focus in the peridomicile in an unused wood pile and collected 12 specimens (one female adult, nine fourth-instar nymphs, and two fifth-instar nymphs) (23°36′39.0″ S, 46°47′14.8″ W). These insects were examined and they tested negative for *T. cruzi*. Two mongrels lived in the backyard. The space where the colony was found was used by one of the animals as a shelter. On examining the eating habit of the triatomine using the enzyme-linked immunosorbent assay technique, it was found to be reactive to dog blood. The house was built of plastered bricks, without cracks. In the rear of its exterior, it had a space of approximately 5 m^2^ with a barbecue grill. This place was partially covered by a roof of Roman tiles and provided access to the backyard, an approximately 10 m^2^-sized area with low vegetation and some fruit trees. The rear part of the property overlapped a fragment of the preserved Atlantic Forest. This forest had been cleared by the irregular settlements of people. The presence of *P. megistus* in the MRSP and, more specifically, in the municipality of Taboão da Serra, occurred from September to April (Figure 2).

**FIGURE 2:**
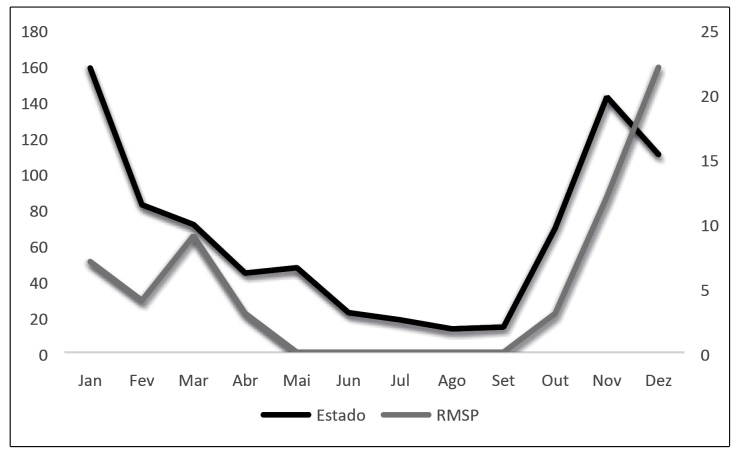
Seasonality of *Panstrongylus megistus* in the State of São Paulo and in the Metropolitan Region of São Paulo, Decade 2010.

*Panstrongylus megistus* is a native species whose adaptation to the domiciliary environment is directly related to humans’ action on the environment and to the reduction in its food sources. In São Paulo, this species presents a restricted distribution range, in which its survival is favored by the rainfall regime, greater humidity, and the type of vegetation cover associated with marsupials of the Didelphidae family, resulting in a high rate of natural infection. This species can colonize the human environment and maintain the circulation of *T. cruzi* in its area. It is important to highlight that the presence of Didelphis spp*.* in the urban areas of the region is quite high. This species has adapted to the urban environment, where it finds shelter and food. 

The seven captured adults of *Didelphis marsupialis* at Point A showed infection in 42.8% of them; this explained the high infection rates by *T. cruzi* found in the collected specimens. These wild animals are likely to maintain the circulation of *T. cruzi* in the area. 

The role of *Didelphis* spp*.* must be highlighted, as high rates of infection by the protozoan as well as persistent parasitemia and infection of the anal glands have been found, of which the latter shows that it can transmit the parasite by non-habitual paths^7^. In the MRSP, opossums (*Didelphis* spp*.*), popularly known as *saruês*, have presented epidemiological importance associated with the presence and infection of triatomines of the species *P. megistus*. Opossums have been very close to humans, invading habitations in search of shelter and food. The presence of opossums in the linings of houses is common.

At another location in the municipality, at a 2-km-distance from these occurrences, the first focus of the species had been detected, associated with a dog shelter in the peri-domiciliary region. This finding demonstrated the species’ potential for occupying artificial ecotopes and adapting to them. Only the adult bug captured after the notification tested positive for *T. cruzi.* In the municipality of São Paulo, *T. cruzi* had already been isolated from this species, which may indicate that the circulation of the protozoan may occur at different parts of the MRSP^8^.

It should be emphasized that, in the artificial environment, domestic animals such as cats and dogs are important links between the peridomicile and the intradomicile. The presence of infected dogs in a house can quadruplicate the infection risk in children^9^. Dogs are a common source of blood for triatomines as they preferably feed on them than on humans^10^.

The destruction or modification of the natural habitat can be associated with the displacement of the species, which migrate to residences where there are domestic animals. This can influence the occurrence and transmission of *T. cruzi*, as it enables the contact between vectors, animals, and humans. Dias *et al*. evaluated the spatial distribution of triatomines in an urban area in the southeast region of Brazil and found that human modification and occupation in natural areas can play a role in the presence of triatomines in the urban environment^11^.

Authors diverge on the domiciliation of the vectors of Chagas disease; some consider it simple opportunism caused by a scarcity of natural food sources whereas others believe that it is a gradual adaptation process subject to natural selection^12^ ^,^ ^13^. However, the invasion of triatomines inside domiciles and the colonization by the species is worrying, as these insects are potential sources of natural infection by *T. cruzi*. Moreover, the combined action of demographic growth and technological development can generate changes in diseases and vectors, posing risks to the human population. 

The presence of *P. megistus* in the state of São Paulo occurs in all months of the year, with periods of higher frequency. The seasonality of the species in the MRSP matches that found in the State, with a predominance in the hottest period of the year, which has the highest rainfall. The association between the collected triatomines and the ones that tested positive indicates that one in every 2.3 captured triatomines is positive for *T. cruzi*. In view of these results, it is possible to argue that the registers show a gradual process of urbanization of the species that must be considered in future vector control actions. Native vegetation guarantees the presence of the wild cycle, and the proximity of these areas to human habitations allows the passive transport of triatomines to enable their movement through ecological corridors. The physical and biological characteristics of the landscape of rural areas are similar to those of native vegetation inside the city.

Although the possibility of vector transmission of Chagas disease in these urban areas is very small, it cannot be ignored. If infected, triatomines that invade homes, to which they are attracted by various factors including light, can transmit *T. cruzi* to humans and domestic animals. It is also important to consider the risk of oral transmission, which is the most frequently observed form of transmission in the Amazon region of Brazil^14^ ^,^ ^15^.

Constant notifications of triatomines have put health surveillance on alert, and different action strategies have been implemented in the MRSP for triatomine control. For example, the prompt response to the notification within 30 days from the collection of the insect by the dweller. In addition, the entomological research radius has been extended, taking into account the vector’s flight amplitude, and educational activities about the vector, the disease, and the collection and sending of suspicious insects have been provided to the population. 
